# Molecular Plasticity of E-Cadherin and Sialyl Lewis X Expression, in Two Comparative Models of Mammary Tumorigenesis

**DOI:** 10.1371/journal.pone.0006636

**Published:** 2009-08-13

**Authors:** Salomé S. Pinho, Celso A. Reis, Fátima Gärtner, Mary L. Alpaugh

**Affiliations:** 1 Department of Carcinogenesis, Institute of Molecular Pathology and Immunology of the University of Porto (IPATIMUP), Porto, Portugal; 2 Department of Pathology, Institute of Biomedical Sciences of Abel Salazar (ICBAS), University of Porto, Porto, Portugal; 3 Department of Pathology, Medical Faculty, University of Porto, Porto, Portugal; 4 Department of Biology, The City College of New York, New York, New York, United States of America; New Mexico State University, United States of America

## Abstract

**Background:**

The process of metastasis involves a series of steps and interactions between the tumor embolus and the microenvironment. Key alterations in adhesion molecules are known to dictate progression from the invasive to malignant phenotype followed by colonization at a distant site. The invasive phenotype results from the loss of expression of the E-cadherin adhesion molecule, whereas the malignant phenotype is associated with an increased expression of the carbohydrate ligand-binding epitopes, (e.g. Sialyl Lewis ^x/a^) that bind endothelial E-selectin of the lymphatics and vasculature.

**Methodology:**

Our study analyzed the expression of two adhesion molecules, E-cadherin and Sialyl Lewis x (sLe^x^), in both a canine mammary carcinoma and human inflammatory breast cancer (IBC) model, using double labelled immunofluorescence staining.

**Results:**

Our results demonstrate that canine mammary carcinoma and human IBC exhibit an inversely correlated cellular expression of E-cadherin and sLe^x^ within the same tumor embolus.

**Conclusions:**

Our results in these two comparative models (canine and human) suggest the existence of a biologically coordinated mechanism of E-cadherin and sLe^x^ expression (i.e. molecular plasticity) essential for tumor establishment and metastatic progression.

## Introduction

Metastasis is classically defined as the lymphatic or haematogenous (blood-born) dissemination of cancer cells from the primary tumor (origin) to lymph nodes and/or to distant sites in the body [1]. The process involves a complex series of steps and interactions between the tumor cells and the microenvironment [2], [3]. The terminal step of this sequential process is colonization of the tumor cells at distant sites in the body. The tendency of aggressive cancers to metastasize is the cause of mortality from the disease [4]. Determining the molecular mechanisms of metastasis is of the utmost importance in an attempt to manage and treat cancer.

Although all cells of the tumor embolus arise from the same parent cell, the embolus is composed of a heterogeneous population of cells. Even in the most malignant primary tumor not all cells have the capability of successfully colonizing at a distant site [5]. This diversity, as well as the molecular and cellular mechanisms underlying the metastatic potential of the tumor embolus result from inherent (clonal selection) or acquired (adaptive) traits or both of the tumor cells composing it [6], [7]. Only a portion of those heterogeneous cells progress to the malignant phenotype and even a smaller portion can successfully colonize, and form deadly metastases [6]. The ability of those tumor cells to conform to new environments is due to a molecular plasticity. Among the events involved in molecular plasticity, loss and gain of key adhesion molecules appears to be a significant factor [8], [9]. Key alterations in adhesion molecules are known to dictate progression from the invasive to malignant phenotype followed by colonization at a distant site [10].

E-cadherin and Sialyl Lewis x (sLe^x^) are two adhesion molecules that govern malignant progression. E-cadherin is an adhesion molecule that plays a key role in homotypic cell-cell adhesion, being classically considered a potent invasion/tumor suppressor gene [9], [11]. Furthermore, sLe^x^ antigen is a carbohydrate structure that is involved in selectin-mediated adhesion of cancer cells to vascular endothelium and this determinant is thought to be closely associated with haematogenous metastases of cancer [12], [13]. sLe^x^ not only is a marker for cancer but also is functionally implicated in the malignant behaviour of cancer cells [14], [15]. Overall, the loss and gain of expression of these molecules are not aberrant variations, but adaptive mechanisms of the cancer embolus, which can defeat our present treatment modalities.

Naturally occurring cancers in canines and humans share many features, including histological appearance, tumor genetics, molecular targets, biological behaviour and response to conventional therapies [16]. Mammary carcinomas, specifically, occur among all taxonomic groups, and comparing the disease in canine models with breast carcinoma in women could greatly improve our understanding of the molecular biology underlying the process of mammary tumorigenesis and progression [17].

In this report we demonstrate that canine mammary carcinoma and the highly metastatic inflammatory breast cancer (IBC) in women, exhibit an inversely correlated expression of E-cadherin and sLe^x^ in cells of the same tumor embolus. Both mammary carcinoma models support a coordinated expression between the two adhesion molecules, suggesting a dynamic transition state or molecular plasticity promoting tumor survival and dissemination during the metastatic progression.

## Materials and Methods

### Ethics Statement

All experiments were performed in compliance with the Memorial Sloan-Kettering Cancer Center Animal Care and Use Program (Protocol Number 06-04-006).

### MARY-X Xenograft and in vitro spheroids

MARY-X was established from a patient with inflammatory breast cancer (IBC) [18]. *In vivo*, MARY-X recapitulates the human IBC phenotype of extensive lymphovascular invasion of the tumor cell emboli. The *in vitro* MARY-X spheroids are prepared by mincing the extricated tumor followed by an overnight incubation under standard tissue culture conditions. The IBC cells are released as sheets and small spheroids initially. Over a period of time, roughly 12–14 hours, the IBC cells as *in vivo*, begin to overexpress E-cadherin and form tight, compact spheroids in suspension. The compact, multicellular in cell suspension spheroids are further purified by partitioning through various size (100 µm, 70 µm and 40 µm pore size; BD Biosciences) cell strainers to produce homogenous preparations of spheroid sizes of 40 µm–70 µm, 70 µm–100 µm and 100+ µm in diameter. Spheroids were maintained in minimal essential medium (MEM) containing 10% fetal bovine serum and antibiotics (100 U/ml penicillin and 100 µg/ml streptomycin) at 37°C in an air-5% CO_2_ atmosphere at constant humidity.

### Canine mammary carcinoma cell line, CMT-U27

The canine mammary carcinoma cell line CMT-U27 was obtained from the Uppsala University, Sweden. CMT-U27 cell line was derived from a primary tumor (infiltrating ductal carcinoma) [19] and when inoculated in the fat mammary pad of female *nude* mice metastasizes to the lymph nodes, lungs, liver and heart. CMT-U27 was cultured in RPMI 1640 with Glutamax medium (Gibco, Invitrogen), supplemented with 10% fetal bovine serum (Gibco, Invitrogen) and 50 µg/ml gentamicin (Gibco, Invitrogen). Culture was maintained at 37°C in a humidified 5% CO_2_ atmosphere.

### Tissue specimens

Ten tissue samples of primary canine mammary carcinomas of different histological types (3 Complex carcinomas; 3 Tubulopapillary carcinomas; 2 Carcinosarcoma; 2 Solid carcinomas) and 3 correspondent lung metastases were used in this study. Accordingly with Peña L et al., canine inflammatory mammary carcinoma (IMC) once diagnosed is involved with other histological types of canine mammary carcinoma including solid carcinomas, tubulopapillary carcinomas and sarcomatous type [20]. The same is also true for feline IMC where the histology revealed types of tubulopapillary mammary carcinomas [21]. In addition, ten *in vivo* (primary tumor and metastases in SCID mice) and ten *in vitro* specimens were harvested from the MARY-X model. The specimens were fixed in 10% neutral buffered formalin. After dehydration and paraffin wax embedment, 4 µm thick sections were cut from each representative paraffin block for staining with haematoxylin and eosin and for double-staining immunofluorescence.

### Double-Labelling Immunofluorescence

For simultaneous visualization of sLe^x^ and E-cadherin on the same tissue section of the two comparative models, MARY-X and canine mammary gland carcinomas, double-label immunofluorescence technique was performed.

Briefly, paraffin sections were dewaxed, rehydrated and then treated with Extran (Merck, Frankfurt, Germany) 0.05% in distilled water for 10 min in a microwave oven at 750 W. After being left to cool for 20 min at room temperature, slides were rinsed twice in Phosphate-buffered saline (PBS), and then incubated for 20 min in a humid chamber with rabbit non-immune serum at a dilution 1∶5 in PBS containing 10% bovine serum albumin (BSA). Sections were incubated with the first primary monoclonal antibody (mAb), anti-human E-Cadherin (clone 36, BD Biosciences Pharmingen, diluted 1∶100 in PBS), overnight at 4°C. After washing twice for 5 min in PBS, sections were incubated with FITC-conjugated rabbit anti-mouse immunoglobulin (code F261; Dako, Glostrup, Denmark; diluted 1∶100 in PBS). Sections were washed twice for 5 min in PBS and blocked with non-immune goat serum diluted 1∶5 in PBS containing BSA 10%. After incubation with mAb KM93 (mouse IgM) diluted at 1∶40, overnight at 4°C, sections were washed twice for 5 min with PBS and then incubated with Texas red-conjugated goat anti-mouse IgM (Jackson Immunoresearch) diluted 1∶50 in PBS. Sections were washed as before and nuclei were counterstained with 4′-6-Diamidino-2-phenylindole (DAPI, Sigma) diluted at 1∶100 in PBS, for 15 min in the dark. Finally, sections were washed twice for 5 min in PBS and mounted with Vectashield (Vector Laboratories, Burlingame, USA).

Dilutions of primary antibodies, secondary antibodies, and DAPI were made with PBS containing 5% BSA.

Immunostained tissue sections were examined under a fluorescence microscope (Leica DMIRE2) equipped with appropriate filters. Separate images for DAPI, Texas-Red and FITC staining were captured digitally at X200 and X400 magnification. The red (for Texas-Red), blue (for DAPI), and green (for FITC) components were merged and combined images were imported into Adobe Photoshop®.

### Immunoprecipitation of E-cadherin from CMT-U27

Cells from CMT-U27 cell line were washed with cold phosphate-buffered saline (PBS) and then lysed with a lysis buffer (1% (v/v) Triton X-100; 1% (v/v) NP-40 in PBS, containing protease inhibitor cocktail (Roche), 100 mM Na_3_VO_4_, 100 mM PMSF) for 15 min on ice. After scraping cell monolayers with a rubber policeman the suspension was centrifuged at 14000 rpm for 10 min at 4°C. Protein concentrations were determined by using a BCA protein assay kit (Pierce).

Total protein (750 µg) of canine cell line was precleared with 50 µl of protein G-sepharose beads (Sigma Aldrich) at 4°C under agitation for 1–2 h to remove non-specific adsorption to the sepharose beads.

After centrifugation at 14000 rpm for 5 min at 4°C, supernatant was incubated overnight at 4°C under agitation with 5 µg of monoclonal antibody against E-cadherin (monoclonal mouse anti human E-cadherin, clone 36, BD Biosciences Pharmingen). Then, incubation with protein G-sepharose at 4°C for 2 h with agitation was performed. Next, the beads were washed three times with an immunoprecipitation buffer (10% (v/v) lysis buffer, 5 mg/ml Na_4_P_2_O_7_, 1% (v/v) NaF 100X in PBS). The immune complexes were released by boiling 5 min at 95°C in Laemmli sampling buffer prior to analyses by Western.

For sLe^x^ carbohydrate detection of E-cadherin immunoprecipitated, nitrocellulose membrane was blocked and then incubated with monoclonal antibody that specifically recognizes sLe^x^ epitope (KM93) with a dilution of 1 µg/ml in PBS with 0.01% Tween 20 (PBS T) with 5% milk.

The membrane was washed three times with PBS T and incubated with HRP-conjugated anti-mouse IgM secondary antibody (Santa Cruz Biotechnology, 1∶3000 diluted in PBS T with 5% milk) during 1 h at room temperature. After washing three times with PBS T, membrane was developed by an ECL according to the manufacturer's recommended protocol.

## Results

### Inversely correlated cellular expression of E-cadherin and sLe^x^ in the MARY-X spheroid

**Figure 1:**
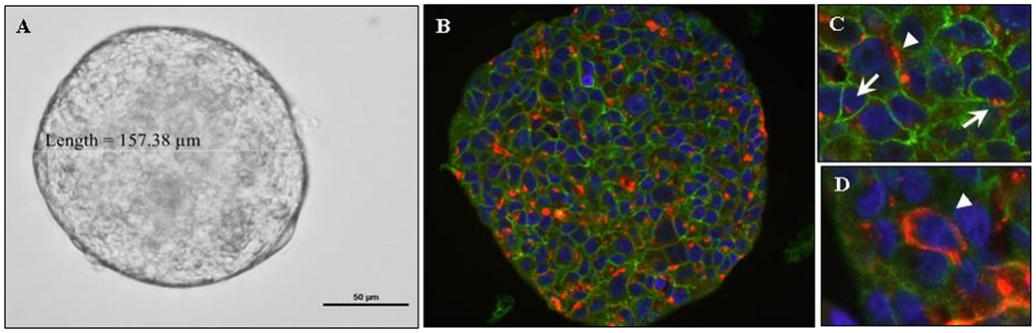
Expression of E-cadherin and sLe^x^ in the MARY-X spheroid. (A) MARY-X *in vitro* spheroid forms a compact, clump of cells due to over-expression of a key adhesion molecule, E-cadherin (light microscopy; 40× magnification). (B) Illustrates a heterogeneous population of cellular expression with a dominant population of cells expressing membrane-positioned E-cadherin (green) and a minority population of cells expressing sLe^x^ (red). (C & D) sLe^x^ was found to be either cytoplasmic- (long arrows) or membrane-positioned (blunt arrows).

MARY-X, like IBC, grows exclusively within the murine lymphatics and blood vessels, which is the signature phenotype of this highly aggressive, malignant breast carcinoma [18]. Both the MARY-X *in vitro* spheroid (Figure 1A) and *in vivo* tumor embolus [data not shown] form on the basis of an intact, over-expressed E-cadherin/α,β catenin axis [22]. Interestingly, MARY-X displays a unique relationship of two key adhesion molecules; over-expression of E-cadherin and under-expression of sLe^x^ confirmed by western blot analysis [23], [24]. This relationship is contrary to what would be expected in a highly malignant carcinoma. To determine if expression levels were due to a homogenous population of cellular expression (i.e. each cell produced excessive amounts of E-cadherin and a limited amount of sLe^x^) double-labeling immunofluorescence of the two adhesion molecules was performed. The majority of cells of the MARY-X *in vitro* spheroid displayed membrane-positioned E-cadherin (green) expression only (Figure 1B). A minority population co-expresses both molecules with a cytoplasmic-positioned sLe^x^ (red) expression (Figure 1C and 1D). An even smaller population of cells, of the MARY-X *in vitro* spheroid display membrane-positioned sLe^x^ (red) expression only (Figure 1D). Co-expression of these two adhesion molecules is rarely seen. Hence the MARY-X spheroid displays an inverse relationship of expression of E-cadherin and sLe^x^. Previous studies indicated a cooperative relationship between E-cadherin and sLe^x^ [23]. However, our study shows that the expression levels are not due to a homogenous population of cellular expression but rather a heterogeneous population of cellular expression. A similar inversely correlated cellular expression between these two key adhesion molecules has also been reported in a malignant canine mammary carcinoma model and was the basis of this study [25].

### Inversely correlated cellular expression of E-cadherin and sLe^x^ throughout the metastatic progression of MARY-X and canine mammary carcinoma

**Figure 2:**
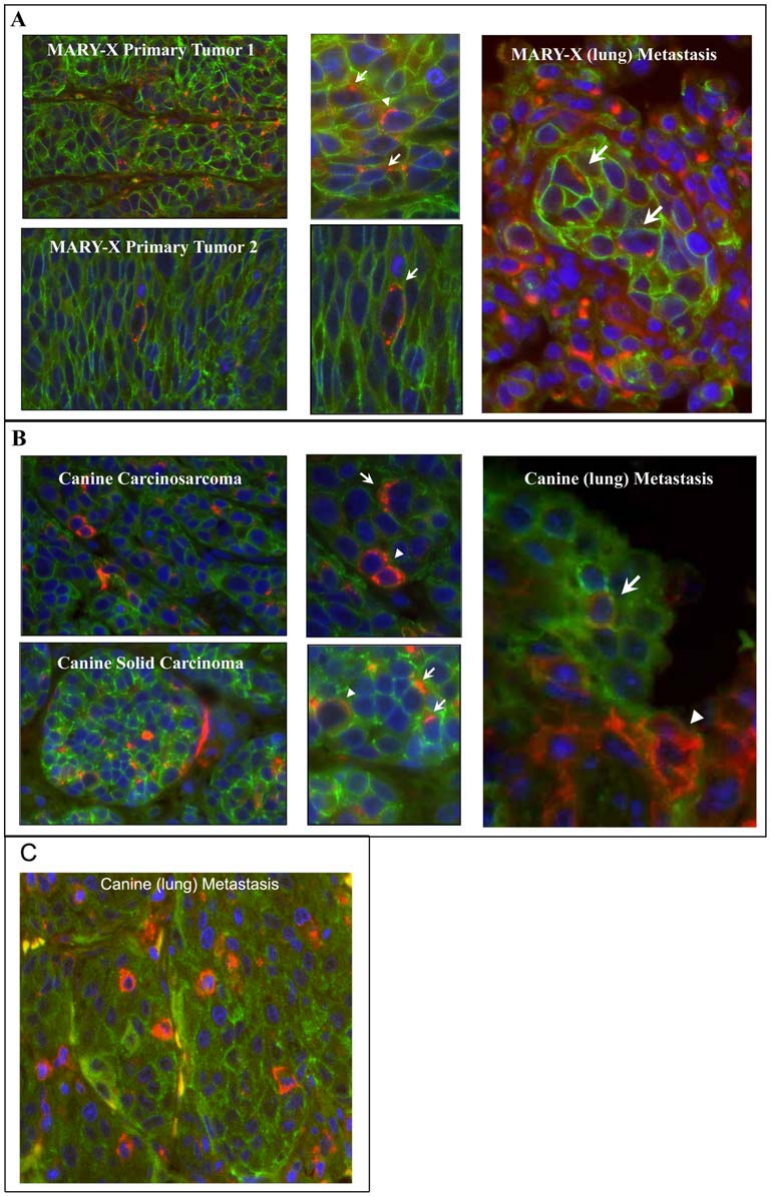
Expression of E-cadherin and sLe^x^ throughout the metastatic progression of MARY-X and canine mammary carcinoma. (A, B) Shows a heterogeneous population of cellular expression in both (A) MARY-X primary tumors and (B) canine primary tumors (carcinosarcoma and solid carcinoma) displaying a dominant population of cells with membrane-positioned E-cadherin (green) and smaller nests sLe^x^ (red) expressing cells (cytoplasmic or membrane-positioned). (A, B; middle panels) Magnified image shows the sLe^x^ found either cytoplasmic- (long arrows) or membrane-positioned (blunt arrows). Mary-X lung metastasis (MARY-X metastasis) displays a dominant population of cells expressing only E-cadherin (green), and a minority population of cells co-expressing cytoplasmic-positioned sLe^x^ (red) (long arrows). (B, C) Canine lung metastasis (Canine metastasis) displays a dominant population of cells expressing only E-cadherin (green), and minority populations of cells either co-expressing cytoplasmic-positioned sLe^x^ (red) (long arrows) or expressing only membrane-positioned sLe^x^ (red) (blunt arrows).

Since adhesion molecules dictate metastatic progression of carcinomas, double labeling immunofluorescence of E-cadherin and sLe^x^ was performed on the primary tumor and corresponding metastases of both the MARY-X and canine mammary carcinoma models to determine the presence of a correlated cellular expression throughout metastatic progression. The majority of the cell population of the primary tumor of both the MARY-X and canine models display a strong membrane-positioned E-cadherin (green) expressing cells with no expression of sLe^x^ (Figure 2A & 2B). To a lesser extent, nests of sLe^x^ (red) expressing cells could be seen throughout both models (Figure 2A & 2B). In the MARY-X model, as seen *in vitro*, sLe^x^ is cytoplasmic-positioned and co-expressed with membrane-positioned E-cadherin (Figure 2A; middle panel). However, a small number of cells display membrane-positioned sLe^x^ expression only (Figure 2A; middle panel). The canine primary tumor is very comparable to MARY-X except that the nests of sLe^x^ expressing cells predominantly display membrane- positioned sLe^x^ only, i.e. no co-expression of the two molecules (Figure 2B; middle panel). The corresponding metastasis of MARY-X and the canine mammary carcinoma also show a dominant population of cells expressing only membrane-positioned E-cadherin (green) (Figure 2A & 2B; right panel and Figure 2C). However, in the few sLe^x^ expressing cells of the MARY-X metastasis co-expression of membrane-positioned E-cadherin with cytoplasmic-positioned sLe^x^ was seen (Figure 2A; right panel). The canine metastasis showed predominantly membrane-positioned sLe^x^ expressing only cells (Figure 2B; right panel and Figure 2C). In the primary tumor and corresponding metastases of both models, co-expression is a rare event. Therefore both models maintain a heterogeneous population of cellular expression through out the metastatic progression displaying an inversely correlated cellular expression of these two key adhesion molecules.

### Sialyl-Lewis X does not mask E-cadherin expression

**Figure 3:**
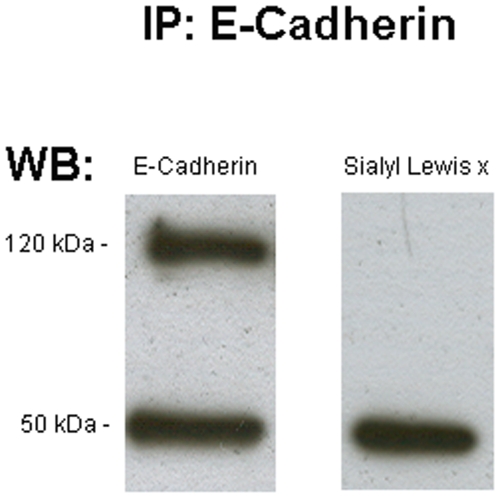
E-cadherin immunoprecipitation of CMT-U27 cell line. The left lane shows the E-cadherin immunoprecipitate. The right lane shows the absence of sLe^x^ in the E-cadherin immunoprecipitate. The 50 kDa bands in both lanes are the immunoglobulin heavy chain.

An E-cadherin immunoprecipitation (IP) was performed in order to rule out the possibility that sLe^x^ immunofluorescense (red) masked E-cadherin immunofluorescense (green) in cells that displayed sLe^x^ expression only (i.e. E-cadherin is a carrier of sLe^x^). This approach was only performed on the canine model due to the greater number of cells that express only sLe^x^ as compared to the MARY-X model. The evaluation of the expression of sLe^x^ using a specific monoclonal antibody showed no detection of this carbohydrate antigen on the E-cadherin immunoprecipitate from canine mammary carcinoma cell line, CMT-U27 (Figure 3). This finding excludes the possibility that the sLe^x^ carbohydrate is attached to E-cadherin.

## Discussion

zTumor cell formation and development of metastasis is a multistep process involving complex interactions between cancer cells, extracellular matrix, lymphovascular system, immune system and target organs [4]. The loss (e.g. E-cadherin) and gain (e.g. carbohydrate ligand-binding epitopes) of key adhesion molecules are contributing factors in the metastatic process [11], [12]. This study investigated two interesting exceptions to the above rule, namely the inflammatory breast cancer model, MARY-X and a canine mammary carcinoma model. Both models are highly metastatic, yet display neither a loss in E-cadherin expression nor overall gain in the carbohydrate ligand-binding epitope of endothelial cells, sLe^x^ [25], [26]. Rather, these two exceptions suggest a more “adaptable” metastatic progression as opposed to a “one way” progression from the invasive (loss of E-cadherin expression) to malignant phenotype (gain in sLe^x^ expression). The heterogeneous population of cellular expression suggests a dynamic “back and forth” transition state between phenotypes that might promote survival of the tumor embolus during metastatic progression.

Our study shows a correlated inverse relationship of E-cadherin and sLe^x^ expression in the genesis, dissemination and subsequent metastases in the MARY-X and canine mammary carcinoma models. These preliminary findings suggest that loss and gain in expression of adhesion molecules is not an aberrant variation but rather an adaptive cellular and molecular mechanism of the cancer that contribute to its metastatic ability. Our findings are the beginning to a better understanding of these adaptive mechanisms. More specifically, since molecular variation contributes to cancer progression and survival then the present findings set the ground for targeting these mechanisms resulting in new therapeutic strategies of metastatic disease. Further work is in progress regarding the clarification of the molecular mechanism underlying gene expression of these two key molecules through out the metastatic progression in both comparative models.

In conclusion, our study suggests the existence of a biologically coordinated mechanism of E-cadherin and sLe^x^ expression (i.e. molecular plasticity) essential for tumor establishment and metastatic progression.
